# An infrastructure for accurate characterization of single-event transients in digital circuits^☆^

**DOI:** 10.1016/j.micpro.2013.04.011

**Published:** 2013-11

**Authors:** Varadan Savulimedu Veeravalli, Thomas Polzer, Ulrich Schmid, Andreas Steininger, Michael Hofbauer, Kurt Schweiger, Horst Dietrich, Kerstin Schneider-Hornstein, Horst Zimmermann, Kay-Obbe Voss, Bruno Merk, Michael Hajek

**Affiliations:** a Institute of Computer Engineering, Vienna University of Technology, Treitlstrasse 1-3, A-1040 Vienna, Austria; b Institute of Electrodynamics, Microwave and Circuit Engineering, Vienna University of Technology,Gusshausstrasse 25/354, A-1040 Vienna, Austria; c Materials Research Group, Helmholtz Centre for Heavy Ion Research (GSI), Planckstrasse 1, D-64291 Darmstadt, Germany; d Institute of Atomic and Subatomic Physics, Vienna University of Technology, Stadionallee 2, A-1020 Vienna, Austria

**Keywords:** Asynchronous digital design, Radiation fault-tolerance, Single-event transients, Single-event upsets, LFSR counters, Elastic pipelines, Muller C-elements, TCAD models, Spice models, SET injection experiments, Ion beam radiation

## Abstract

We present the architecture and a detailed pre-fabrication analysis of a digital measurement ASIC facilitating long-term irradiation experiments of basic asynchronous circuits, which also demonstrates the suitability of the general approach for obtaining accurate radiation failure models developed in our FATAL project. Our ASIC design combines radiation targets like Muller C-elements and elastic pipelines as well as standard combinational gates and flip-flops with an elaborate on-chip measurement infrastructure. Major architectural challenges result from the fact that the latter must operate reliably under the same radiation conditions the target circuits are exposed to, without wasting precious die area for a rad-hard design. A measurement architecture based on multiple non-rad-hard counters is used, which we show to be resilient against double faults, as well as many triple and even higher-multiplicity faults. The design evaluation is done by means of comprehensive fault injection experiments, which are based on detailed Spice models of the target circuits in conjunction with a standard double-exponential current injection model for single-event transients (SET). To be as accurate as possible, the parameters of this current model have been aligned with results obtained from 3D device simulation models, which have in turn been validated and calibrated using micro-beam radiation experiments at the GSI in Darmstadt, Germany. For the latter, target circuits instrumented with high-speed sense amplifiers have been used for analog SET recording. Together with a probabilistic analysis of the sustainable particle flow rates, based on a detailed area analysis and experimental cross-section data, we can conclude that the proposed architecture will indeed sustain significant target hit rates, without exceeding the resilience bound of the measurement infrastructure.

## 1 Introduction

TU Vienna’s FATAL project (Fault-tolerant Asynchronous Logic)^2^ is devoted to the foundations of a framework for modeling and analysis of fault-tolerant asynchronous digital circuits, using fault-tolerant distributed algorithms knowledge. Besides handling circuit/environment specifications, composition and decomposition and hierarchical proofs [1–3], it also addresses adequate failure and metastability models for digital circuits. Overall, FATAL’s aim is to contribute to a “Theory of Dependable VLSI”, which has been identified as a major challenge in [4].

Unlike most research in this area, FATAL is primarily (albeit not exclusively) focusing on asynchronous circuits. This is backed-up by the fact that, ultimately, the operation of any combinational digital logic gate is inherently asynchronous. Moreover, asynchrony is also a quite natural phenomenon at higher system layers, as in *Globally Asynchronous, Locally Synchronous* (GALS) architectures [5], for example. Unfortunately, unlike for synchronous systems, fault-tolerance is difficult to guarantee in asynchronous systems [6], and has hence not received much attention in asynchronous digital circuits [7–10,1,11].

In order to systematically reason about fault-tolerant VLSI circuits, suitable failure models are mandatory. For “classic” sources of errors, like aging and electric wear-out [12], there is a huge body of work to rely upon. For radiation-induced errors, however, which are increasingly dominating the failure rate of deep submicron VLSI circuits [13,14], this is not the case. Radiation-induced errors, collectively termed *single-event effects* (SEEs) in literature, occur when the active area of a VLSI circuit is hit by ionizing particles (or even by neutrons, which typically result from heavy ion interactions with nitrogen or oxygen atoms in the atmosphere).

As opposed to permanent SEEs such as latch-up, threshold voltage shifts and even destructive burn-outs in power semiconductors [15–17], the primary concern in modern VLSI circuits are transient SEEs: An ionizing particle hitting a reverse-biased junction of a transistor deposits charge along its track, which in turn can cause a *single-event transient* (SET) pulse (0.1–1 ns range) at the output. If the affected transistor is part of a storage element (e.g., an SRAM cell or a latch), this may cause the element to flip its state, leading to a *single-event upset* (SEU). Unfortunately, SEUs may also occur if the hit transistor is part of combinational logic, since a sufficiently strong SET may propagate until it reaches a storage element where it is latched. In any case, however, the errors resulting from SEUs are not permanent but rather transient (i.e., can be corrected), and are hence called *soft-errors*.

This paper presents an overview and first results of our approach for developing suitable failure models for radiation-induced transient failures of digital VLSI circuits in FATAL. It is organized as follows.

In Section 2, we survey the current state of the art, while in Section 3, we provide an overview of our general approach. As in [18–20], it primarily rests on two lower-level simulation models, the *3D model* and the *Spice model*. The 3D model is a Synopsis 3D TCAD model of our elementary target circuits, which include (a chain of) inverters, Muller C-elements, etc. Particle hits are incorporated via the SRIM–TRIM nuclear code simulation software [21]. In order to calibrate the 3D model, which obviously also depends on technology parameters like doping profiles, we manufactured our target circuits in 90 nm UMC CMOS technology and performed elaborate analog SET recording experiments under carefully controlled microbeam irradiation at the GSI in Darmstadt [22].

In Section 4, we describe the current version of the Spice model, which consists of the transistor schematic models of our target circuits augmented by Spice models that mimic SET generation in all critical parts. For the latter, we use single-ended injection of a double-exponential current into the source of a transistor [23]. Since Spice model simulations are orders of magnitude faster than 3D model simulations, the Spice model is a suitable tool for the thorough (and reasonably fast) investigation of the SET behavior of any digital circuit made up of an arbitrary combination of our elementary target circuits. This, in turn, is the appropriate basis for devising and calibrating/validating suitable digital radiation failure models in FATAL.

Still, in order to rule out unnoticed modeling errors in the 3D model and/or in the Spice model, and to validate the accuracy of the digital failure model w.r.t. technology-related parameters like transistor sizes and doping profiles, we are currently designing a special digital FRad Chip that will allow us to do long-term digital monitoring of radiation-induced failures in basic asynchronous circuits. Its design and pre-fabrication analysis/evaluation, based on a detailed Spice model of the circuit according to the general FATAL approach outlined above, are the main focus of this paper: In Sections 5 and 6, respectively, we describe and justify our selection of the elementary radiation targets and the measurement architecture to be incorporated in the FRad Chip. Section 7 is devoted to the analysis of the resulting size requirements and the fault-tolerance properties of the measurement architecture, Section 8 provides a simple probabilistic analysis based on cross-section data inferred from our radiation experiments. Backed up by these results, we conclude in Section 9 that we can indeed expect to get meaningful measurement data from experiments using the FRad Chip, which will be an important part of our future work in this area.

## 2 Overview of SET-related research

Dependability concerns have stimulated a large body of research work devoted to fault-tolerance in VLSI. Classically, faults resulting from excessive manufacturing variabilities, aging and wear-out are handled primarily by technological fault prevention methods [12] and/or via fault detection and recovery approaches [24]. Replication-based fault-tolerance methods like *dual* or *triple modular redundancy* (DMR, TMR) have also been implemented, albeit only for very demanding applications.

During the last decades, radiation-induced SEEs became an increasing concern in VLSI, see e.g. [13,20,14,25–28]. Besides permanent SEEs such as latch-up, threshold voltage shifts and even destructive burn-outs in power semiconductors [15–17], this primarily concerns transient SEEs. Formerly, SEEs caused problems only in aerospace applications [16,15], where high-energy particles are abundant due to cosmic rays interacting with the atmosphere. However, the advent of nanometer-technology VLSI has dramatically raised both the circuit complexity and clock speeds and decreased supply voltage and charges [26,13]. As a consequence, overall, modern VLSI circuits show considerably higher *soft-error rates* (SERs) nowadays, despite considerable improvements of process technology. It has hence been reported that, despite the strong decay of charged particles with atmospheric depth, the SERs of chips operated at sea-level are also likely to exceed acceptable limits [14,28].

At sea level, neutrons are the primary source of SEEs in VLSI [29,14,30,31]. In addition, some *α*-induced SEEs are caused by impurities in chip packaging materials, solder balls, etc. [14,32]. Various mitigation techniques, mostly using replication-based fault-tolerance techniques, have been invented to reduce the SER of modern VLSI chips.

### 2.1 SER assessment

Whereas SEUs and SERs in synchronous circuits have been/are heavily studied [14,13,26,28], considerably less research has been devoted to SETs. In particular, very little is known about SET generation and propagation in asynchronous circuits like (chains of) Muller C-elements [33], which is the primary focus of FATAL.

In order to assess the SER of a circuit, a number of different effects must be considered jointly:

*SET generation*: 2D or even 3D physical modeling is used to obtain the charge/current induced in a transistor in a certain technology on a certain particle hit. Several finite element-based simulation tools like DAVINCI [34,35,20], DESSIS [18,36–38], NanoTCAD [19,39], Synopsis SDevice [40,41] and Cadence Sentaurus-Device [42] have been employed for this purpose, also in mixed-mode simulations with Spice models. Apart from “regular” SET generation [27,19,20], physical simulations and experiments also revealed irregular phenomenons [43,44,41]. Note that 3D modeling usually suffers from the unavailability of technology-related data like doping profiles, which are (at best) compensated by calibrating the models, by e.g. using transistor models from manufacturer’s process design kits [38].

Moreover, there are several papers in literature that deal with experimental SET measurements. Suitably designed radiation targets (usually long inverter chains) are exposed to accelerated radiation tests (using neutron [45] or heavy-ion [46–51,39,52,53,44,41,43] beams) and/or laser-based failure injection [46,54,55,49,56,51]. The resulting SETs are measured using several different approaches: Besides indirect approaches based on SER measurements [46,57–60], which use the correlation between SET pulsewidth and the *linear energy transfer* (LET) of specific heavy-ions, there are several different approaches for digital on-line measurement of SET pulsewidths using variable delay latches [48,47] or self-triggered inverter + latch chains [50,55,39,45,61]. A few papers also report on analog measurements of SET pulsewidths by directly connecting a real-time oscilloscope [51,56,49,36]. Our on-chip analog sense amplifiers [22] seem to be the first low-intrusive SET measurement attempt following the latter approach.

*Electrical masking*: Analytic [62–64,37] or Spice models [65,34,46,18,66,19] are used to describe the actual generation of SETs, as well as SET propagation along a chain of gates, in particular, in research on radiation-hardened circuit architectures. Obviously, Spice models can also account for the fact that transistors are not isolated, but rather employed within circuits. In fact, SET pulses depend very much on operating conditions like supply voltage, temperature, load, and driving strength [34,67,18,20,19,37,56,51].

*Logical masking*: Even a strong SET can be masked out in combinational logic, e.g., when arriving at an AND gate with some other input at low. An accurate analysis of logical masking requires an exhaustive classification of all sensitive paths in a given combinational logic [20]. Such an explicit modeling is of course expensive (in [68], it is shown that computing the most reliability-critical path is NP-hard); alternative approaches rely on fault injection [69,18] and probabilistic modeling [66,70].

*Temporal masking*: In synchronous circuits, SETs originating from combinational logic can only lead to SEUs if they propagate to the flip-flops and arrive there within their setup and hold time. Temporal masking is very effective if the latter are small relative to the clock cycle [13,26,66,70,20,28]. However, due to ever increasing clock frequencies, this assumption does no longer hold true, and the problem is further exacerbated by SETs hitting the clock drivers [71].

In asynchronous circuits, temporal masking effects are very different. Actually, temporal masking is tightly intertwined with logical masking here: The ability of an asynchronous circuit like a Muller C-element to memorize a (SET-)transition on some input depends on its other input(s). In reasonably regular structures, like bundled data or delay insensitive asynchronous pipelined architectures, there are ways to analyze temporal masking similar to synchronous circuits [54,10]. In general, however, an accurate analysis of temporal masking in asynchronous systems requires an exhaustive classification of all paths involved in the completion detection process and hence explicit modeling.

To combine all the above issues, hybrid modeling and simulation are quite common: 2D/3D physics + Spice modeling is used e.g. in [35,19,20], and an even more elaborate hierarchical framework based on fault dictionaries is introduced in [18]. To compute the SER of a given chip, simulation [72–74,14], probabilistic analysis [66,73,70,20,26,71] as well as validating measurements [31,58,18,59,66,75,13,61,72,76] have been used.

Obviously, such SER results allow the assessment of technology scaling [72,13,56,75,26]. Essentially, the SER per gate resp. per bit stays constant or slightly increases/decreases (there is no common trend). The total SER per chip, however, has increased dramatically [72,14] due to increasing chip complexity. Although technology improvements (*silicon on insulator* (SOI), *metal–insulator–metal* (MIM) caps, etc.) [77,19] are very effective for mitigating SEEs, they are not sufficient to maintain acceptable SERs. Moreover, they are considered too expensive [14] for replacing bulk technology in general, and there are also reports of unexpected effects like SET pulse broadening during propagation [51].

### 2.2 SER mitigation techniques

Techniques for SEE mitigation are sometimes mandatory for achieving acceptable SERs, even for devices operated at sea level. Besides design hardening techniques (transistor sizing, layout) [19], there is a broad spectrum of mitigation approaches [25]. Most of them are based on “fine-grained” fault tolerance. Examples are the SEU-tolerant *dual interlocked storage cell* (DICE) [78], SET filtering approaches [79,80] in latches, dual-rail data encoding like *null-convention logic* (NCL) [10], or low-level replication [81]. Existing architectural solutions are primarily based on error detection and recovery/reconfiguration: Examples are the popular EDAC-protected memory (EDAC stands for *error detection and correction*) [25], deadlock detection in *quasi delay insensitive* (QDI) asynchronous circuits [82,9], or space-hardened FPGAs [83]. For non-transient SEEs, mitigation techniques e.g. based on current sensors for latch-up detection are also known [25]. Architectural fault-tolerance solutions based on DMR/TMR [7,8,84] or ingenious time redundancy techniques [85] also exist.

One important effect that defeats most existing SET/SEU mitigation techniques are *multiple upsets* (MUs) and charge sharing effects [44]. In addition to the obvious fact that billions of (vulnerable) deep submicron transistors on a single chip make multiple hits at different locations quite likely [86], there is also an increased probability that a single particle hit upsets multiple near-by transistors at the same time [72,59,76], or even generates multiple SETs [41]. Besides statistical techniques [87] for extracting MU statistics from SEU data in SRAMs, such data have also been obtained via direct measurements [72,74,59]. Interestingly, it has been observed that, unlike SEUs, MUs are very much directional-dependent in anisotropic radiation fields [88]. Although advanced VLSI technology like SOI decreases the sensitivity to MUs [76,13,19,77] as well, the problem cannot be mitigated by such technological means alone. However, we are not aware of alternative mitigation technique for MUs (besides higher-redundancy EDAC in memory arrays).

Particularly relevant in the FATAL context is the (relatively sparse) existing work on fault-tolerant asynchronous circuits. Asynchronous circuits have both advantages and disadvantages with respect to fault-tolerance: All existing asynchronous approaches are inherently tolerant to (most) delay faults [54], but are susceptible to deadlocks [82] and, even worse, to superfluous transition generation [54]. Moreover, with respect to SETs, one may expect that general asynchronous circuits intrinsically have a higher SER since they may not benefit much from temporal masking effects. Several papers deal with SET/SEU sensitivity analysis of asynchronous circuits: QDI circuits and mitigation techniques are dealt with in [8,54,89,84,9], NCL-based approaches are considered in [10,90], and bounded delay solutions are dealt with in [7]. For QDI circuits, we note that [54] describes a tool-based approach for sensitivity analysis. In addition, [9] investigates SET propagation over state-holding elements in asynchronous circuits, and introduces a design for fail-stop QDI asynchronous pipelines that can deal with both SEUs and standard stuck-at faults.

Backed up by the above overview, we conclude that, despite the large body of work on radiation-induced errors in VLSI, the available data is not sufficient for defining and validating failure models for fault-tolerant asynchronous digital circuits.

## 3 FATAL general approach

The ultimate goal of FATAL is *not* the faithful and detailed modeling of the generation and propagation of SETs/SEUs in a circuit (which is the traditional approach, see e.g. [66]), but rather the development of a *digital failure model* that focuses on the *observable behavior* of a digital circuit under radiation, at some chosen level of detail (see e.g. [65] for an early example of this approach). Nevertheless, developing a meaningful digital failure model primarily requires means for validating and calibrating the failures predicted by the model for certain operating conditions (which of course also include radiation field characteristics) w.r.t. the actual failures observed in a real chip under these conditions.

As validating and calibrating candidate digital failure models cannot reasonably be done by means of experiments with real circuits, due to lacking controllability and excessive measurement times and efforts, we use an accurate Spice model for this purpose. Spice models are in fact very convenient and hence widely used in practice for similar goals, cp. [18–20], as they combine excellent controllability with reasonably small simulation times. Our Spice model consists of the analog models of our target circuits, which include e.g. (a chain of) inverters and Muller C-elements (see Section 5), augmented by additional Spice models that mimic SET generation in critical transistors. For the latter, we use standard single-ended injection of a double-exponential current [23] into the transistor sources, see Section 4 for details. As witnessed by the pre-design analysis of our FRad Chip (see Section 7), the Spice model is indeed a suitable tool for the thorough (and reasonably fast) investigation of the SET behavior of any digital circuit made up of an arbitrary combination of our elementary target circuits.

In order to provide meaningful results, however, it is absolutely instrumental for the Spice model to faithfully and accurately model SET generation in a real circuit under given operating conditions. For the same reasons as for the validation and calibration of digital failure models, however, namely, lacking controllability and excessive measurement times and efforts, we could not use experiments for calibrating the Spice model either. This problem is solved by using another model, the 3D model, which finally *can* be validated experimentally.

### 3.1 The 3D model

**Fig. 1:**
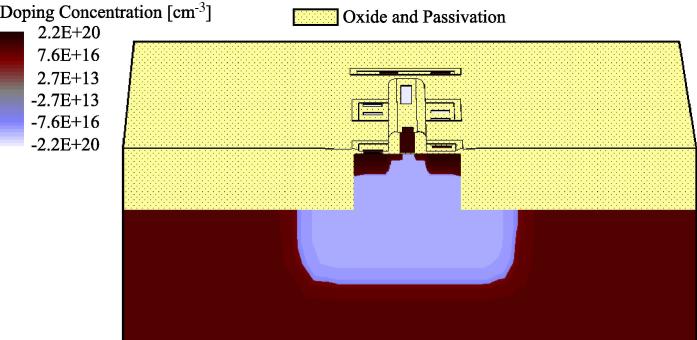
TCAD 3D Structure of an inverter (cutting plane through NMOS).

Like in [18,19,38,20], we use 3D device simulations for calibrating and validating the Spice model. Relying on 3D models of our elementary circuits, which are derived from the detailed floorplan of a circuit in a given technology, these simulations allowed us to accurately determine their behavior under heavy-ion radiation: More technically, the charge generation along the ion track in the silicon is modeled with the SRIM–TRIM nuclear code simulation software [21]. The resulting charge generation profile is then used as an input for the Synopsis TCAD device simulator, which can compute accurate SET current and voltage pulses. As a typical example, Fig. 1 shows the 3D TCAD model of a single inverter structure. In order to reduce the necessary number of mesh points, the metal connections are not included in the 3D model but rather considered using proper boundary conditions. This does not adversely affect the quality of the simulations results, but saves a lot of simulation time.

Unfortunately, accurate 3D device simulation also needs technology-related information, like doping profiles and well depths: The SET generation process is very sensitive to those parameters. Since such information is usually only known to the manufacturer (and typically not disclosed to customers), the need arose to also calibrate and validate the 3D model. Rather than using transistor models provided in the manufacturer’s *process design kits* (PDK), as done in [38], which are of questionable use for accurately calibrating the complex SET generation process, we conducted carefully controlled SET measurements [22] at the microprobe facility at the GSI [91] in Darmstadt (Germany) for this purpose. A microprobe facility allows a very accurate (sub-μm) deposition of *single* ions, with well-known energy, at any location on the chip.

**Fig. 2:**
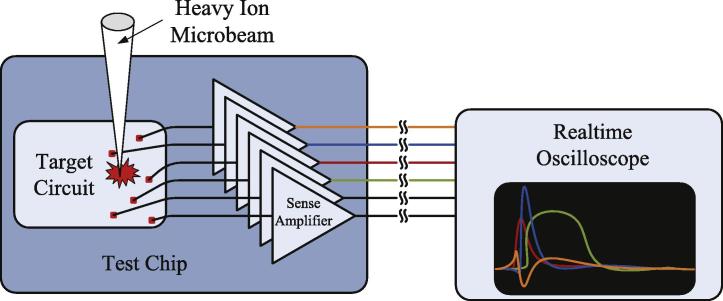
Test chip schematics for calibration and validation of the 3D model and the spice model.

Our radiation experiments were performed with custom ASICs fabricated in 90 nm UMC CMOS technology. They integrate some of our target circuits (inverter chains and Muller C-elements) with very high-speed analog sense amplifiers, as shown in Fig. 2. The SET voltage pulses generated by the target circuits upon an ion hit (which is detected by a channeltron at the microprobe facility) are low-intrusively captured by the sense amplifiers and finally recorded by an external real-time oscilloscope.

**Fig. 3:**
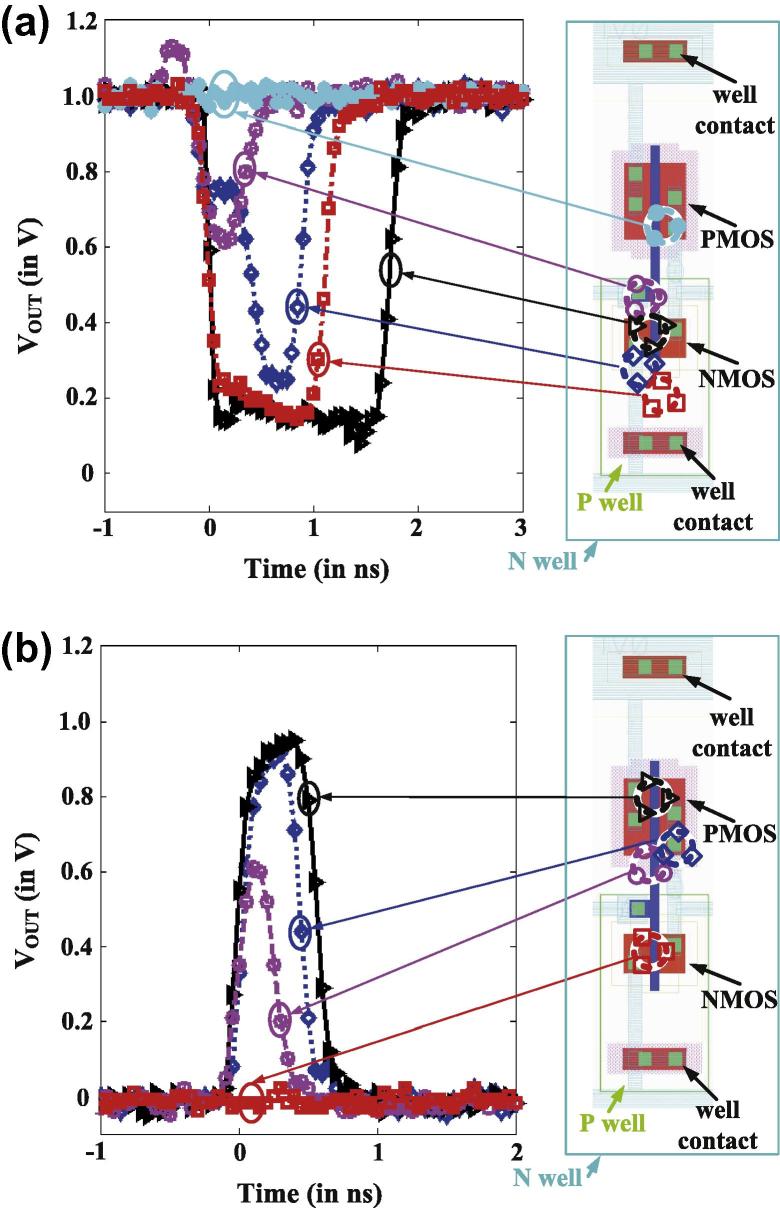
Measured SET voltage pulses (inverter) under heavy-ion (^197^Au, 946 MeV) irradiation [22]: SETs for (a) low (0), (b) high (1) inputs.

For the inverter chain target circuit, we measured typical voltage pulses as shown in Fig. 3 for different impact positions of the ions. It is important to mention that the resulting SETs do not only depend on the impact position of the ion but also on the state of the circuit: For the two different inverter input levels, quite different behavior could be observed [22]: *full-width half-maximum* (fwhm) pulse widths of up to ∼1.6 ns for a low (0) input level, and up to ∼800 ps for a high (1) input level were observed, with very small rise times.

Finally, calibration of the technology-dependent model parameters of the 3D model was done by means of offline comparison of the SET voltage pulses predicted by the 3D device simulations and the actual SET voltage pulses recorded in our experiments. Our efforts resulted in 3D model predictions that match the experimental data sufficiently well,^3^ such that we could use the predicted collected amount of charge in the source contacts of the transistors for calibrating the Spice model as described in Section 4.

More specifically, we could infer from the 3D model that the amount of collected charge strongly depends on the impact position of the ion. Approximately 300–350 fC are collected by the source contact for worst case scenarios, resulting in SETs which are capable to propagate. The corresponding rise times of the SET voltage pulses at the output of the simulated inverter are in the range of 10–70 ps.

### 3.2 The FRad Chip

Recall that the ultimate goal of FATAL is a digital failure model, rather than the 3D model and the Spice model (which are—albeit being of considerable independent interest—only intermediate “tools” here). In order to (i) spot possibly remaining modeling errors in the digital failure model, and (ii) validate the accuracy of the digital failure model w.r.t. technology parameters like transistor sizes and doping profiles, we designed a custom ASIC called *FRad Chip*, which combines a suitably rich target circuitry (made up of our elementary circuits) and an elaborate digital measurement unit. Rather than analog SET recording on a real-time oscilloscope [49], as used for validating our 3D models, the FRad Chip incorporates only digital measurement circuitry that continuously observes and counts radiation-induced failures occurring in the target circuits. Unlike for SEUs, where bit-flips in SRAM memory arrays are a convenient means for collecting SEU statistics, see e.g. [16], we are not aware of any comparable circuit in the literature. Its description and pre-fabrication analysis/evaluation using the general FATAL approach outlined above are the primary focus of this paper.

Ultimately, the FRad Chip will provide us with a means for the “end-to-end validation” of the accuracy of digital failure model predictions by means of (long-term) radiation experiments.

## 4 The Spice model for SETs

A number of Spice models have been proposed in literature over the years, which model radiation hits via current injection. Most proposed models agree in the qualitative definition, but differ in essential quantitative aspects [92]. Roche et al. [93] only considered peak currents, which is not realistic for the time-varying restoring current, and also overestimates the critical charge *Q*_*crit*_. These issues have been addressed to some extent by Xu et al. [94], by defining *Q*_*crit*_ with respect to the static tripping point of an SRAM cell; it fails to consider the corporate dynamics of voltage transients at the struck node, however. Zhang et al. [95] estimated *Q*_*crit*_ in terms of transistor parameters and injected currents, characterized by magnitude and duration, but observed a discrepancy w.r.t. reality. One of the reasons is the use of a suitably matched rectangular current pulse, instead of an exponential one. Actually, so far the most agreed model to mimic the actual charge deposition mechanism of a particle strike uses double exponential currents [96,38,42], so we adopt it for our analysis as well.

More specifically, for injecting SETs in a transistor of our target circuits, we use a current source connected to the source of the transistor that generates a double-exponential current pulse according to the following equation [97]:

(1) $$I_{P}(t)=I_{0}(e^{-t/T_{\alpha }}-e^{-t/T_{\beta }})\text{.}$$

Herein, *I*_*P*_ denotes the transient current pulse, *I*_0_ the peak current of the two exponential terms, *T*_*α*_ the decay time (fall time) of the current pulse, and *T*_*β*_ the time constant for initially establishing the ion track (rise time). Easy calculations reveal that the total charge *Q*_*P*_ of such a pulse is

(2) $$Q_{P}(t)=\int_{0}^{\infty }I_{P}(t)\mathrm{dt}=I_{0}(T_{\alpha }-T_{\beta })\text{,}$$

whereas the peak current of the SET (*I*_*peak*_) is given by

(3) $$I_{\mathrm{peak}}=I_{0}\left(e^{\frac{T_{\beta }\log(T_{\beta }/T_{\alpha })}{T_{\alpha }-T_{\beta }}}-e^{\frac{T_{\alpha }\log(T_{\beta }/T_{\alpha })}{T_{\alpha }-T_{\beta }}}\right)\text{.}$$

All our simulation experiments were conducted using HSPICE and Cadence Spectre simulators.

We executed a number of initial simulations using an inverter chain as our target circuit for calibrating the parameters *I*_0_, *T*_*α*_ and *T*_*β*_. Our goal is to determine a parameter setting which leads to SET voltage pulses (resp. critical charges *Q*_*crit*_) that mimic the ones measured (resp. predicted by the 3D model) according to Section 3.1 as faithfully as possible. Recall that the measured SET voltage pulse lengths ranged up to 800 ps (resp. 1.6 ns) for logic high inverter input (resp. low input), with *Q*_*crit*_ in the range of 300–350 fC. We varied the current model parameters *I*_0_, *T*_*α*_ and *T*_*β*_ until both (a) *Q*_*crit*_ = *Q*_*P*_ according to Eq. (2) and (b) the SET voltage pulse lengths predicted by Spice matched reasonably well.

**Fig. 4:**
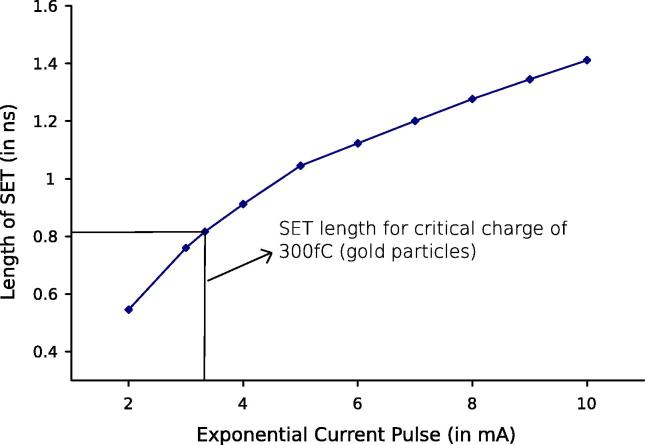
Length of SET vs. exponential peak current *I*_0_.

In order to get some basic understanding of the transformation of an analog current pulse in an inverter to a digital voltage pulse at the output, Fig. 4 plots the length of the SET voltage pulse observed at the buffered inverter output over *I*_0_, for fixed^4^ *T*_*α*_ = 100 ps and *T*_*β*_ = 10 ps; this results in a peak current of *I*_*peak*_ = 0.78*I*_0_ according to Eq. (3). There is already a digitally visible SET with a length of 125 ps for *I*_0_ = 1 mA, while for *I*_0_ between 2 mA and 10 mA the length of the observed digital SET grows (approximately logarithmically) from 540 ps to 1.4 ns. For *I*_0_ = 3.33 mA, (a) Eq. (2) reveals an injected charge of about *Q*_*P*_ = 300 fC, matching our *Q*_*crit*_, and (b) Fig. 4 reveals an SET voltage pulse length of 800 ps that is also in the right order of magnitude.

Indeed, fine-tuning of the model parameters provided us with maximum SET voltage pulses of 800 ps for a high inverter input, which nicely matches our measurement results. For a low inverter input, the maximum SET duration we could generate with our choice of model parameters is 980 ps, which somewhat underestimates our longest measured SET durations. We conjecture that this is, in part, an artefact of the lack of parasitic capacitance in the Spice model of the target circuits taken from UMC’s PDK. It may also be a sign of the need of some structural improvement of the double exponential current model (cp. [66,19]), however.

It must be noted here that there are inherent fundamental differences between 3D model and Spice model that render a *perfect* matching of the pulse shape ultimately impossible: The Spice model shall represent the complex charge deposition (and collection) process, which is—apart from the characteristics of the particle impact as such—determined by various and highly non-linear “current paths”, by a single current source with a more or less pre-determined current shape, which provides only a few parameters for tuning. When applying such a substantially simplified model to (purposely) abstract away details, one cannot expect a perfect representation of reality. The 3D model, on the other hand, is much more powerful in this respect, but its high computational complexity makes it practically impossible to also incorporate the entire relevant “context” of the hit transistor in the circuitry (which, however, determines the transformation of the current pulse into a voltage pulse and its propagation).

The single-source double exponential current model employed in our Spice model represents the current state-of-the-art, which has been considered a suitable tradeoff between tractable complexity and sufficient accuracy in most of the related research work. For compatibility with this research, and also due to lacking alternatives, we simply had to accept the artefacts mentioned above. However, part of our envisioned future work in this area will be devoted to alternative Spice models, which provide better modeling accuracy with still acceptable complexity.

## 5 Selection of radiation target circuits

The specific purpose of the FRad Chip leads to the following design requirements and constraints:

• There is no need to capture analog effects.
• The transformation of analog effects to the digital domain obviously influences digital observations. In order to be as realistic as possible, this transformation shall solely be performed by the target circuits (implicitly).
• In order to get relevant results, we need to select target circuits that are elementary and/or frequently used in practice.
• To explore the behavior of target circuits both in dynamic and steady state, we need the option of exercising/stimulating the targets when exposed to radiation.
• We want to apply sources of radiation whose characteristics is similar to what circuits are typically exposed to in practice. These sources have in common that (a) time and location of particle hits are not controllable, (b) the particle hit rate is reasonably low, and (c) the target has to be brought into some environment (e.g., a radiation chamber) that is spatially confined. This substantially impacts the design of the overall measurement infrastructure.

Considering these issues, we have decided for a set of target circuits that will be detailed below.

### 5.1 Inverter chain

**Fig. 5:**
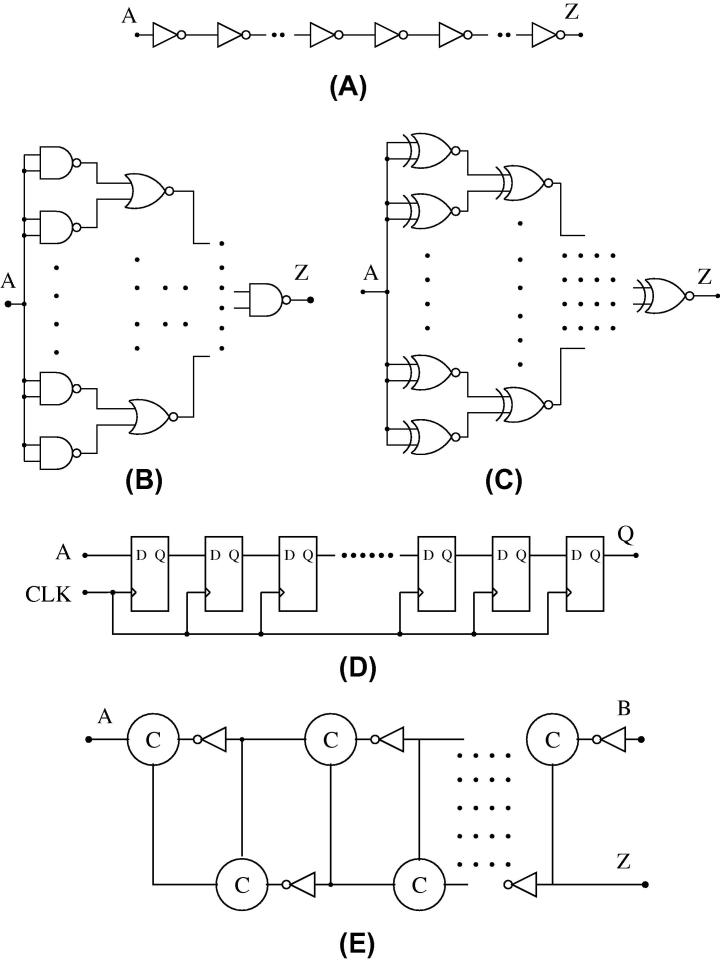
Gate level schematics of target circuits.

Inverters are the most basic and simple elements in CMOS and thus the preferred radiation targets in the existing literature. For a given transistor sizing, they are also the fastest CMOS circuits, thus potentially vulnerable to even very short SETs. Incorporating inverters in our study is hence not only relevant in practice, but also allows us to compare our results to literature. In order to have a reasonably large target area and a rich testbed for investigating propagation effects, we provide a chain of 17 inverters, as shown in Fig. 5A (in fact, multiple instances thereof).

### 5.2 NAND–NOR tree

NAND trees have always been a topic of interest in terms of testing and SETs. NAND and NOR implementations are slightly more complex than inverters and therefore exhibit different phenomena. One is the availability of two inputs, which allows us to form a tree structure. Another one is the possibility of logical masking of errors.

We have 64 NAND gates in the first stage that are all driven by a single input signal, which allows us to jointly stimulate activity with minimal overhead. The outputs of the NAND gates fan-in as inputs for NOR gates and so on, yielding a tree with 127 gates in total, see Fig. 5B. The tree has seven levels, hence in the fault-free case the output will be the inverse of the input.

### 5.3 XNOR tree

The XNOR gate forms the equivalent of the logical OR for transitions, another very basic functionality in asynchronous circuits and also fundamental for parity checking circuits. Compared to NAND/NOR gates, it has a very different internal structure. We employ an XNOR gate implementation based on a CMOS transmission gate with inverter.

We again use a seven level tree structure. It is apparent from Fig. 5C that two outputs of XNOR gates fan-in to an XNOR gate at the next level. The inputs of the 64 XNOR gates in the first stage are again all connected to a single input. Due to the XNOR function all gates will therefore, independently from this input, present a logic 1 at their outputs, and the tree output will transiently go to low only in case of an SET somewhere in the tree. As we will see, this behavior is very convenient for our purposes.

### 5.4 Flip-flop chain

Flip-flops are the fundamental building blocks of virtually every synchronous design. Like inverters, they have hence received much attention in radiation-related research in the past, which will allow us to compare our results against existing data. As shown in Fig. 5D, we provide a chain of 33 edge-triggered master–slave D-flip-flops, which are implemented using transmission gates and inverters.

### 5.5 Elastic pipeline

**Fig. 6:**
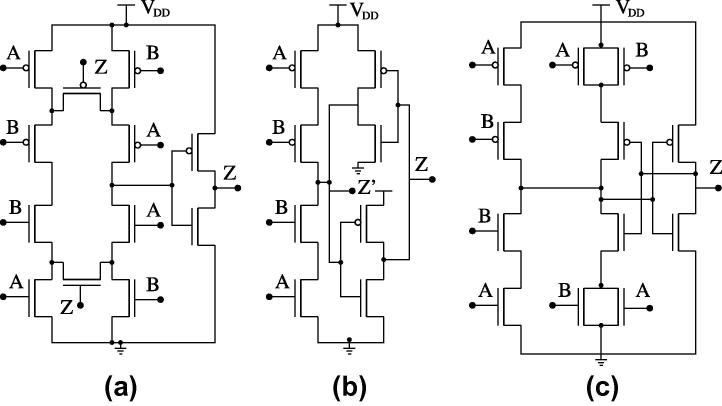
Transistor level schematics of: (a) Van Berkel, (b) weak-feedback, (c) conventional Muller C-elements.

An elastic pipeline is built from Muller C-elements and inverters as shown in Fig. 5E. A Muller C-element is *the* fundamental building block in the design of self-timed digital circuits [98]. Although it can be viewed as a (combinational) AND for transitions, it is a state-holding element much like an asynchronous set-reset latch. Three different CMOS implementations have become popular, all of which we will use as target circuits:

(a) CMOS implementation introduced by Van Berkel [99], see Fig. 6a.
(b) CMOS implementation using an inverter latch introduced by Martin [99], see Fig. 6b.
(c) Conventional pull-up pull-down CMOS implementation introduced by Sutherland [99], see Fig. 6c.

The elastic pipeline in Fig. 5E is essentially a FIFO buffer for signal transitions that is often used in handshake-based circuits. The C-elements in the pipeline propagate the signals in a carefully controlled way that maintains the integrity of each wave [99,100]. The speed of signal propagation is determined by the actual delays of the circuit.

The most interesting property of the circuit is that it is delay-insensitive, i.e., it works correctly regardless of wire and gate delays. Since many asynchronous designs are based on elastic pipelines, its behavior in the presence of radiation effects (SET generation, propagation and latching) is of utmost relevance. As we will see later on, beyond being an attractive target, elastic pipelines are also useful as measurement circuits.

## 6 Measurement architecture

The measurement architecture of the FRad Chip must facilitate the continuous monitoring and recording of all occurring SETs, at the level of digital signals, in statistical long-term experiments. To get as much information as possible from an experiment, as many nodes in our target circuits (abbreviated DUT, for *device under test* in the sequel) as possible must be monitored simultaneously. At the same time, the number of monitored nodes is limited by the available die area and the number of pins of the FRad Chip.

On-chip preprocessing is used to reconcile these requirements: (i) We extract SET occurrences out of the possibly superposed dynamic operation of the DUTs as early as possible. (ii) Since we are not interested in the precise time of occurrence of SETs in statistical analysis, it suffices to just count the number of SETs in consecutive *measurement periods*, at the end of which the counts will be transferred to some off-chip data recording unit and be reset. (iii) To save pins, the data transfer will be performed serially after parallel/serial conversion (PISO). (iv) Since DUTs and on-chip preprocessing circuitry compete for the same die area, the latter must be as lean as possible.

**Fig. 7:**
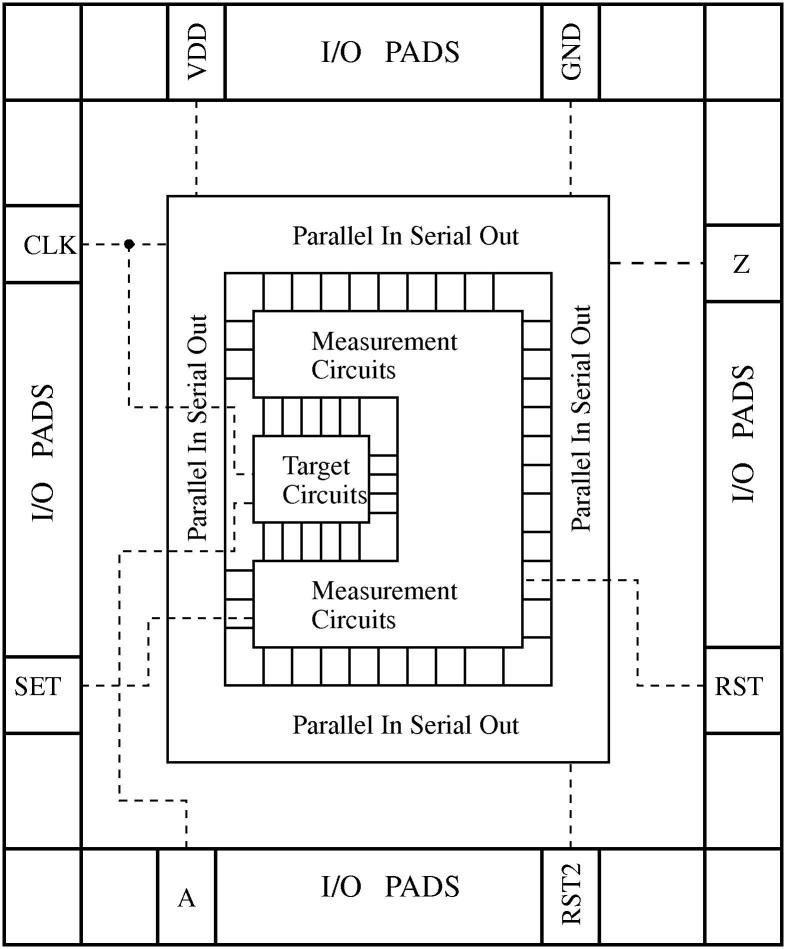
Structure of the chip.

Fig. 7 shows the structure of the resulting FRad Chip architecture. Notice the strict separation between target circuits, measurement circuits and PISO that may turn out helpful in specialized ion-beam experiments, in which radiation hits can be restricted to a certain area.

**Fig. 8:**
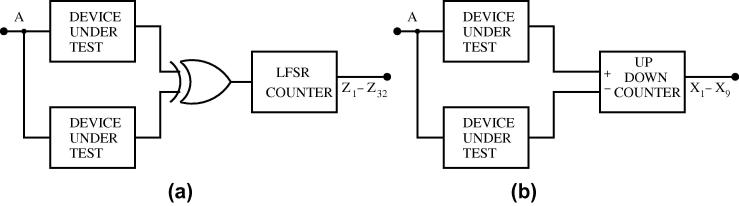
Extracting SET occurrences from the observed activity by (a) XOR or (b) difference counter.

We want to investigate SET generation in our DUTs both in static and in dynamic mode. For the latter, we provide a common data signal that can be used to collectively stimulate switching activity. Recall that the XNOR tree has the beneficial property of exhibiting activity at its output only in case of a particle hit. Therefore, it is sufficient to use a simple incrementer for counting SETs here. Unfortunately, not all our other DUTs exhibit this nice behavior. Since the generation of the stimuli is under our control, however, we can easily provide a correct reference signal for comparison/subtraction purposes. This may be achieved by a simplified version of the DUT (even a simple wire or an inverter does the job, since all our DUTs except the XNOR tree exhibit a behavior equivalent to that of a wire or inverter) or by another instance of the same DUT. In principle, any mismatch between the DUT output and the reference signal can be extracted by an XOR, whose output feeds the SET counter, see Fig. 8a. The problem here is that the XOR tends to produce glitches in case of a non-zero skew between DUT and reference, thus potentially leading to spurious counts. Considering that we are not interested in the exact *temporal* matching of the behaviors of DUT and reference, but rather in matching their signal traces, a more appropriate solution is an up/down counter, with the DUT output feeding one input and the reference signal feeding the other one, see Fig. 8b. Obviously, we cannot use a synchronous up/down counter, since SETs would not adhere to setup/hold constraints and hence cause metastability. Moreover, in order to catch even short SETs, our counters should be as fast and sensitive as possible. Fortunately, there is a nice and area efficient way of building an asynchronous up/down counter for transitions that is based on an elastic pipeline. Fig. 11c illustrates its principle.

Alternatively, we may use an incrementer as well to count the transitions performed by the DUT during a measurement period. However, in this case, we will see the sum of transitions due to the SETs in the DUT plus those due to the regular DUT switching activity. As the stimuli applied to the DUT are under our full control, we can subtract the latter a posteriori; the incrementer must accommodate a much larger count value, though.

Being on-chip, the measurement circuitry will be exposed to radiation just like the DUTs and hence has to operate properly in the presence of particle hits. Recall that the FRad Chip is not primarily designed for being used under microbeam irradiation, but rather with any radiation source. There are many options available for making a circuit tolerant against these single, uncorrelated particle hits, such as TMR [101], coding [102], time redundancy [103], or radiation hardening [79]. However, all of those tend to cause high overheads (at least 200%), thus rendering a pretty large share of the die area unusable for additional DUTs. Considering that both proposed types of counters resemble interesting target structures by themselves (namely, a flip-flop chain as well as an elastic pipeline), we decided *not* to mask particle hits in these circuits but rather to let them occur: This effectively turns the on-chip measurement infrastructure into an additional radiation target. The remaining challenge is, however, to find a clever arrangement that allows us to distinguish between errors that occurred in the original target circuit and those in the counters. To this end, we use the following three strategies:

• For our SET counters, we employ a *Linear Feedback Shift Register* (LFSR) instead of a simple incrementer. The important benefit of doing so is that the counting sequence in a (carefully chosen) LFSR always involves multiple bit changes per count, hence a single bit flip caused by an SEU will lead to a dramatic change in the count sequence that is easily recognizable by an a posteriori analysis.
• To make sure that we have a correct copy of the count available even in case of a counter hit, we use duplication. Since, thanks to using an LFSR counter, we can identify the corrupted value, there is no need to go for triplication.
• For the difference counter, we cannot rely on recognizing erroneous counts. Duplication just allows us error detection but not recovery. A viable alternative is using an up/down counter in combination with an LFSR counter (which must be quite wide then, of course). This will not only allow recovery of the correct count, but will also provide diversity that might turn out very beneficial in a radiation environment.

**Fig. 9:**
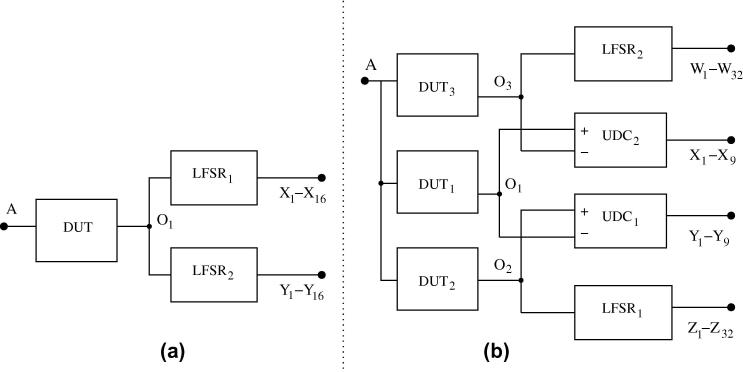
Proposed measurement architectures for (a) XNOR tree; (b) all other target circuits.

Fig. 9 shows the finally chosen architectures. For the XNOR tree, we simply use two LFSR counters in parallel, as shown in Fig. 9a. Although we expect only few hits per DUT in a measurement period on average (see Section 8), we decided to go for a 16-bit LFSR (for details see below) in order to retain a sufficiently long counting sequence; this makes the recognition of incorrect counts more reliable. By using two LFSR counters, we make sure that we have a correct count available in case one LFSR has been hit.

For all the other target circuits, we use the architecture shown in Fig. 9b. It comprises three DUTs of the same type, which we mutually use as a reference. For example, the behavior of DUT_2_ is observed by the two up/down counters UDC_1_ and UDC_2_. Note that these counters have different references (DUT_1_ and DUT_3_, respectively) and use different polarity (UDC_1_ counting down and UDC_2_ counting up on output transitions of DUT_2_). In principle, this architecture allows us to tolerate any of the two up/down counters becoming faulty. However, as we cannot be sure to safely recognize every SEU of an up/down counter, it may (in rare cases) happen that we end up with two counts indicating different numbers of SETs, which without additional information are both plausible.

For DUT_1_, we use a different strategy: Its behavior is observed by both UDC_1_ (counting up) and an LFSR counter. The benefit here is that, as motivated above, we can trust to recognize any faulty behavior of the latter. So in case the LFSR counter indicates a plausible number of SET occurrences in the target, we can simply trust it, while otherwise we still have the result of UDC_1_ as a backup. Here we need a 32-bit LFSR for reasonably long measurement periods without wrap-around (42 s for a 100 MHz input data stimulus), which we consider necessary for a safe recognition of counter hits. Finally, we use the same strategy for DUT_3_.

Given the relatively low hit rate (according to Section 8, we will tune measurement period and radiation intensity to experience only a few hits per period), our general strategy in interpreting an observed scenario is to assume the lowest possible number of hits that could have led to the given observation. Considering, e.g., that UDC_1_ counts up for failures in DUT_1_ while it counts down for those in DUT_2_, one might argue that SET observations may cancel out each other. This is, however, not the case, since we have redundant information in UDC_2_ and the LFSR counter. With this combined information, it is possible to accurately identify every single hit, all double hits in both the target and the measurement circuits, and even many multiple hits correctly (for details see Section 7.5). Backed up by the probabilistic calculations in Section 8, we are convinced that our architecture represents an excellent choice with respect to the combined criteria of area efficiency, fault tolerance, diagnosability and diversity. Overall, it clearly surpasses the more evident solutions using three LFSR counters or three up/down counters.

In the following we will present some details of our SET counter implementations.

### 6.1 Linear Feedback Shift Register (LFSR) based counters

An LFSR is a synchronous shift register with XOR gates forming selected feedbacks [104], which produces a deterministic and periodic pseudo-random counting sequence. It is heavily used in practice for generating CRC checksums and pseudo-random bit strings. Compared to conventional binary counters [105], an LFSR reduces the amount of required logic and minimizes routing complexity. With feedbacks forming a “maximum length polynomial”, an LFSR with *n* flip-flops can implement a 2^*n*^ − 1 state counter [104,106]. Two circuit structures can be used to implement a given polynomial, namely, the many-to-one design and the one-to-many design.

**Fig. 10:**
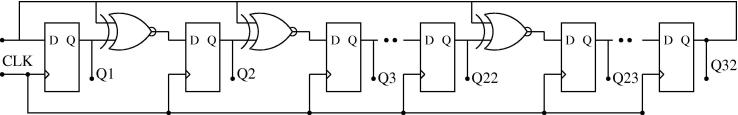
32-bit LFSR.

We selected the 32-degree polynomial *x*^32^ + *x*^22^ + *x*^2^ + *x* + 1 shown in Fig. 10 and the 16-degree polynomial *x*^16^ + *x*^14^  + *x*^13^ + *x*^11^ + 1 for our measurement architecture. We chose a one-to-many design based on XNOR gates for both, since the associated count sequence involves many bit changes per step, which is beneficial for detecting a single bit fault.

### 6.2 Up–down counters

**Fig. 11:**
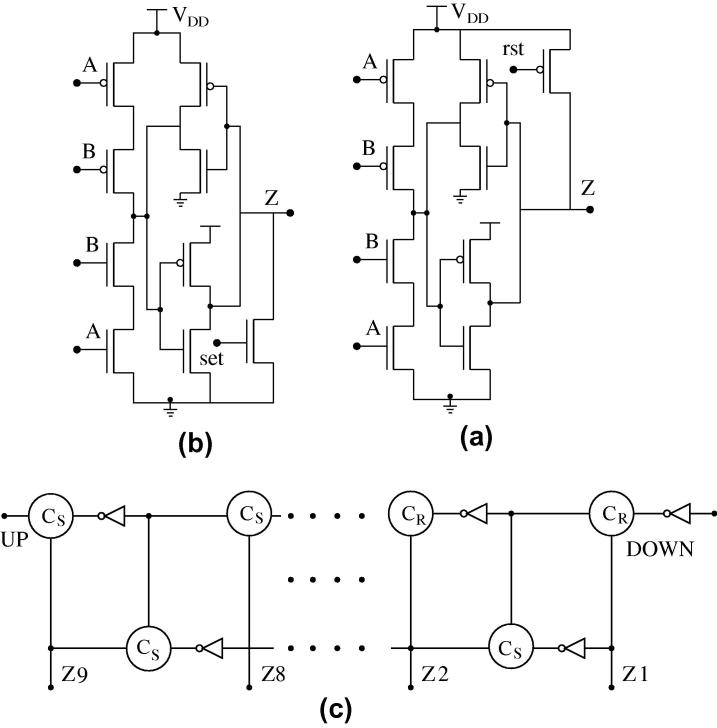
Schematic of (a) Muller C-element with reset (rst), (b) Muller C-element with set (set) and (c) up/down counter.

Our SET up/down counters will be implemented as 9-stage pipelines made up of Muller C-elements [107] with weak feedback inverters. Inputs *A* and *B* in Fig. 5E are used as up and down count inputs, connected to the DUTs; the output *Z* is not used. In order to enable the possibility of counting down, we preset the pipeline to a value of 5 upon reset. For this purpose, we need to add extra transistors (with appropriate sizing) to the C-elements as shown in Fig. 11a and b.

The up/down counter utilizing the two versions of the C-elements (with set and rst) and inverters is shown in Fig. 11c. The outputs *Z*1–*Z*5 are preset to 1, while *Z*6–*Z*9 are preset to 0; all bottom-row C-elements are initialized to 0. A transition on *UP* will add to the transitions already present in the pipeline, while a transition at *DOWN* will remove one transition from the pipe, thus decreasing the count.

## 7 Evaluation and analysis

The goal of this section is to provide an overview and some results of our pre-fabrication analysis of the proposed measurement architecture of the FRad chip. Apart from area considerations, our primary concern is an evaluation of the resilience against particle hits.

### 7.1 Overhead analysis

Table 1 lists the total number of transistors required by the different target circuits described in Section 5 and the SET counters introduced in Section 6.

The FRad Chip hosts three instances of each target circuit, two 32-bit LFSR counters and two up/down counters for every measurement setup. The exception to this is the XNOR tree target, one instance of which is monitored by two 16-bit LFSR counters. The resulting area consumption and the overhead incurred by the measurement circuits over the target circuits are given in Table 2. Note that the only substantial overhead incurred by the measurement setup occurs for the inverter chains, which is due to the small size of the target. For the other target circuits, the measurement overhead is very reasonable. On average, the measurement circuitry consumes 19% more area (in fact fewer transistors) than the target circuits. Given that our SET counters can also be seen as additional target circuits in our architecture, the overhead is acceptable.

### 7.2 Fault-tolerance analysis setup

The primary tool for the analysis of our measurement circuits’ resilience against particle hits is simulation-based fault injection, using appropriate Spice models as described in Section 4. To get confidence in our architecture, we injected faults in each and every gate of each SET counter and analyzed the resulting behavior of the circuit.

We used release 5.10.41 of the Cadence Virtuoso Front-end to Back-end design environment to create the schematics of our circuits. They were all designed using UMC 90 nm NMOS and PMOS device models. We chose custom *W*/*L* (width/length) ratios for the NMOS transistors, while the *W*/*L* ratios of the PMOS transistors were chosen based on the structure of the corresponding circuit. The Spice netlists were extracted from the respective Cadence schematics.

We performed all our analog simulations using HSPICE Version D-2010, using the following setup: To generate switching activity in the circuits, we toggled the data input every 5 ns. After 10 ns, we triggered the set and reset signals of the counters for about 40 ns, which initializes the LFSR counter to 0 and the up/down counters to 5. At specifically selected times during normal operation, we triggered SETs by injecting a current pulse in the Spice netlist (refer to Section 4).

### 7.3 LFSR counter evaluation

The regular operation of the 32-bit LFSR is illustrated in Table 3: With each rising clock edge, the counting proceeds by one step; the 32-bit LFSR will step through a sequence of about 4.2 billion different values. A low at the RST input resets the count value to 0.

For our fault-tolerance analysis, we injected faults in each of the XNOR gates and flip-flops independently. Selected results are listed below (see also Table 4):

• Injection of an SET causing a bit flip from 1 to 0 in the XNOR gate tapped between *Q*_1_ and *Q*_2_ (please refer to Fig. 10) at 75 ns: Here the benefit of using an LFSR for counting becomes apparent. While only one bit of the output actually changes due to the SET, the related change in the counting sequence is drastic and hence easily recognizable: According to Table 3 the value following 29,360,146 should be 62,914,594, but here it is 29,360,144, effectively causing a huge jump in the counter sequence (see rightmost column).
• Injection of an SET causing a bit flip from 0 to 1 in the flip-flop with output *Q*_15_ at 80 ns: This increased the LFSR count by 2^15^ and the actual count by 1.64 billion steps approximately. Again this is easy to detect.

Overall, this confirms that a single bit flip in the LFSR counter is witnessed as billions of skipped transitions. Due to our careful selection of the LFSR polynomial, its one-to-many implementation (see Section 5), and backed up by numerous further experiments, we can indeed generalize this observation: A single bit flip in any LFSR cell will always infer a much larger and hence easily detectable effect in the actual count, which finally justifies our decision to use an LFSR counter.

### 7.4 Up/down counter evaluation

In our analysis, SETs were injected into all C-elements and inverters to evaluate the resulting behavior of the up/down counter introduced in Section 6. Table 5 lists some of the scenarios obtained (e.g. at 75 ns and 105 ns). Recall that the up/down counters are initialized to a count of 5, represented by 111110000 on *Z*_9_ ⋯ *Z*_1_ in Fig. 11c. A fault injected at 130 ns in the C-element that drives the output *Z*_8_, e.g., changed the outputs *Z*_6_, *Z*_7_ and *Z*_8_ to 1. There were also many instances when the fault injected at the same node in a different time interval just changed the output *Z*_8_ temporarily to 1 (for one step) and switched back to 0.

Overall, we observe that the effect of an SET in an up/down counter is dependent on the location and the direction of the resulting bit flip. Unlike in the LFSR case, the initial effect of the fault is not “amplified”, such that a particle hit in the up/down counter cannot easily be distinguished from a regular counting step caused by an SET in the associated target. This confirms that some kind of replication is indeed mandatory for using these counters in our measurement architecture.

### 7.5 Overall measurement architecture and fault dictionary

We have created a comprehensive fault dictionary for our measurement architecture, which associates every fault scenario (single or multiple SET hit (s) in counters and targets) with its “syndrome”, i.e., the set (*U*_1_, *U*_2_, *L*_1_, *L*_2_) of resulting readouts on the up/down counters UDC_1_ and UDC_2_, as well as the LFSRs 1 and 2. Used in the reverse direction, this dictionary allows us to infer from an observed syndrome the fault scenario that caused it, with, e.g. (*U*_1_, *L*_2_, *D*_2_) indicating that UDC_1_, LFSR_2_ and DUT_2_ have been affected by an SET. This mapping, unfortunately, is not bijective, as different multiple-fault scenarios may map to the same syndrome. We use two strategies to handle this issue: (1) By carefully choosing the measurement period (see Section 8), we can safely neglect the probability of experiencing many SET hits within one period (i.e., before reading out and re-initializing the counters). This allows us to ignore fault scenarios involving more than, e.g., four SETs in our dictionary. The same reasoning supports our strategy (2), namely, associating an observed syndrome with the scenario that involves the lowest number of faults, as it is far more probable to occur than other matching scenarios that might exist. Of course, however, this can lead to misinterpretation in rare cases.^5^

Table 6 shows an excerpt of our fault dictionary. Herein “∗” and “X” both indicate an incorrect counter value, with the latter being recognizable as an error and the former not. “√” indicates that the expected LFSR value for the fault free case is read, “+” stands for a correctly incremented LFSR value.

It turns out that our architecture facilitates correct identification of the hit circuit for all single faults. The same is true for all double faults (not shown for brevity). Furthermore, most of the triple faults and even quadruple faults are correctly identified; the few problematic cases that lead to a wrong interpretation are shown in the table. In the case when all four counters are hit, we do not have any useful information left, of course.

### 7.6 SET simulations of the measurement infrastructure

**Fig. 12:**
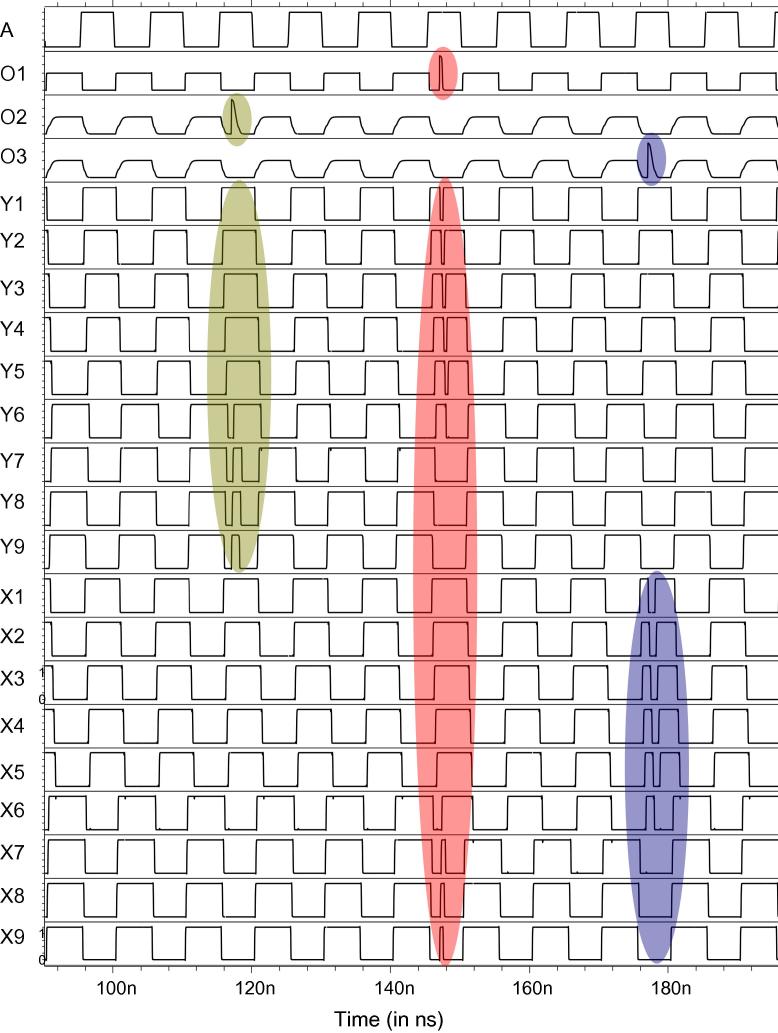
Spice simulation of the up/down counters.

**Fig. 13:**
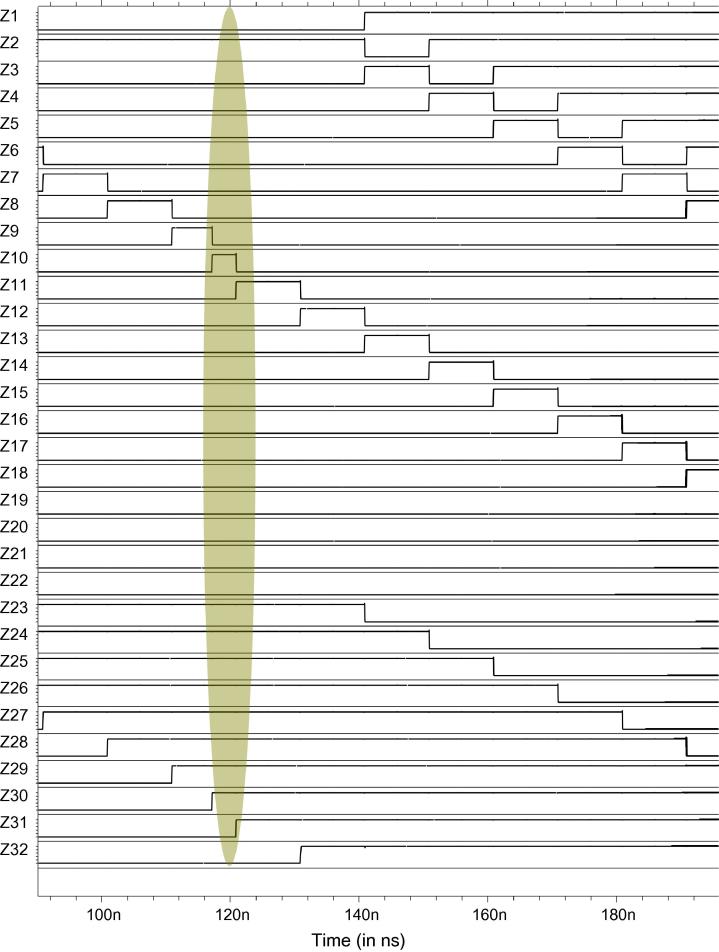
Spice simulation of the LFSR counter 1.

**Fig. 14:**
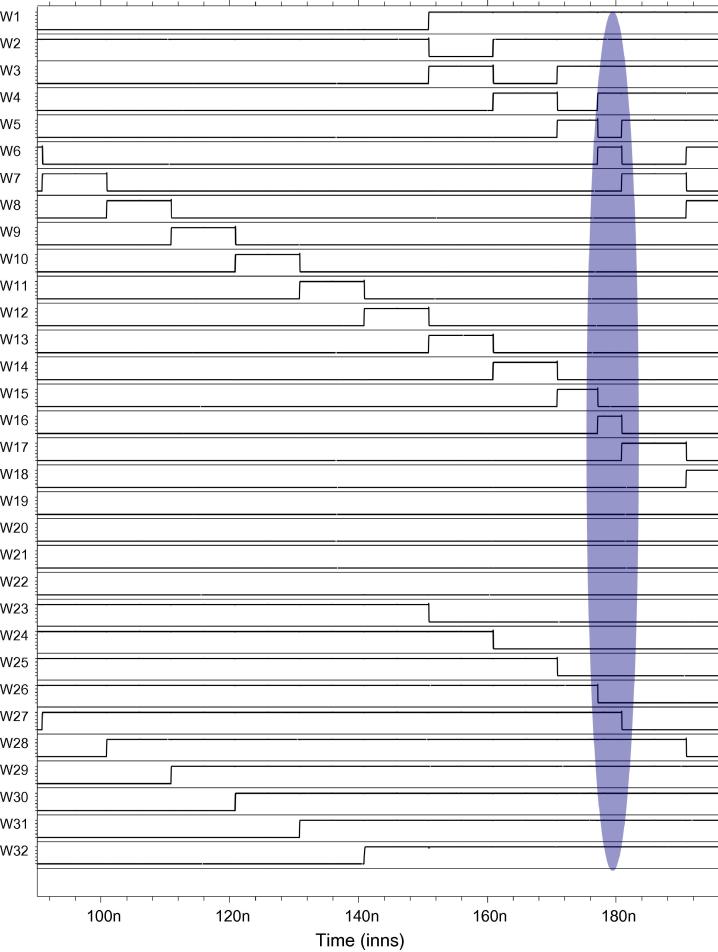
Spice simulation of the LFSR counter 2.

To give a brief overview of our Spice analysis performed to validate our architecture, we present an example considering an inverter chain as the DUT, using the measurement architecture from Fig. 9. We injected SETs in all the three target circuits DUT_1_, DUT_2_, DUT_3_ at different times, observable at outputs O_1_, O_2_, O_3_ in Fig. 12: An SET is injected at 117 ns in DUT_2_, at 147 ns in DUT_1_ and at 177 ns in DUT_3_. The effect of these SETs in the up/down counters and the LFSR counters can be inferred from Figs. 12–14.

More specifically, the effect of the SET injected at 117 ns can be observed in UDC_1_ (refer to signals Y1–Y9 of the UDC in Fig. 12) and in LFSR_1_ (refer to Fig. 13). The effect of the fault injected at 147 ns in DUT_1_ can be observed in UDC_1_ and UDC_2_ (refer to signals Y1–Y9 and X1–X9 in Fig. 12). Similarly, the fault injected at 177 ns in DUT_3_ can be observed in UDC_2_ (refer to signals X1–X9 in Fig. 12) and in LFSR_2_ (refer to Fig. 14). One notices that, at 190 ns, the SETs injected in the target circuits cancelled the counts of UDC_1_ and UDC_2_, thus bringing them back to the initial state. However, the effect of the SETs in DUT_2_ and DUT_3_ is still observable in LFSR_1_ and LFSR_2_, respectively.

More specifically, assuming that these were the only three SETs observed in this target circuit during the measurement period of 40 s, we will observe no change in the UDCs and one extra count in both the LFSRs at the end of the measurement period. From these values, we can infer that the faults did not occur in LFSR_1_ or LFSR_2_: If a fault occurred in the LFSR, then there would not be just one extra count but millions of extra counts. From the LFSR’s values we can thus infer that the fault occurred in the target circuits DUT_2_ and DUT_3_ and thus explain the SET’s effect in the UDCs: Based on their values, we deduce that the SET in DUT_1_ cancelled the effect of SETs created by DUT_2_ and DUT_3_ in the UDCs. This is how we determined the corresponding entry in the fault dictionary in Table 6.

Our fault dictionary has been validated by means of numerous simulated SET injections (up to seven at a time) into various locations, using the same process as explained above. We are hence convinced that the chosen measurement architecture will indeed work as expected.

## 8 Probabilistic analysis

Given the non-negligible number of transistors *I*_*M*_ of the measurement circuitry *M* as compared to the number of transistors *I*_*T*_ of the target circuitry *T* in Table 2 in Section 7 on one hand, and the ability of *M* to tolerate just a double hit for sure^6^ on the other hand, the question about feasible measurement periods *Δ* = *Δ*(*ϕ*) for a given particle flux *ϕ* (in particles per μm^2^ s) arises: *Δ* must be chosen small enough such that, with reasonably high probability, there are at most two hits in *M* during *Δ*; we call such a measurement period *safe*. At the same time, with reasonably high probability, two consecutive hits in *T* should occur within some *P* safe measurement periods sufficiently often, in order to get statistically meaningful data on the SET generation process.

A gross estimate of *Δ* and *P* can be determined using cross section data. Although such an estimate necessarily ignores the fact that target and measurement circuitry have very different structure and topology, it provides meaningful results due to the fact that we do not rely on *single-event-upset* (SEU) cross sections but rather on SET cross sections: Whereas it is known that memory elements like flip-flops are more susceptible to radiation than combinational logic, this is primarily a consequence of the fact that SETs in combinational logic are relatively unlikely to be latched. Consequently, they do not as easily lead to an SEU as SETs resulting from a direct hit of a flip-flop. By contrast, the SET generation process is the same both in combinational logic and in flip-flops.

Our radiation experiments for validating the 3D model provided a (saturated) SET cross section *γ* of about *γ* = 5 μm^2^ for our 90 nm ASIC technology, which matches the figures given in the literature [52]. The cross section expresses that a total fluence of 1 particle per *γ* results in 1 SET per device of interest (in our case, per transistor) on average. Trivial calculations based on expected values reveal that if we choose $\phi \Delta =\frac{1}{\gamma I_{M}}$, we get one hit in *M* during *Δ* on average. Since *Δ* should be chosen large enough to fully exploit *M*’s double-hit resilience, but should only rarely lead to triple hits, we choose

$$\Delta =\frac{C}{\gamma \phi I_{M}}\text{,}$$

for some constant 0 < *C* ⩽ 2, which leads to *E*[*H*_*M*_] = *C* hits in *M* on average. For arbitrary distributions of the number of hits *H*_*M*_ in a single measurement period in *M*, Markov’s inequality *P{H*_*M*_ ⩾ *h*} ⩽ *E*[*H*_*M*_]/*h* reveals a triple-hit probability of *p* = *P{H*_*M*_ ⩾ 3} ⩽ *C*/3; it can be made sufficiently small by choosing *C* sufficiently small. As this results in a geometric distribution of safe measurement periods, we can expect an average of *P*_*M*_ = 1/*p* ⩾ 3/*C* consecutive safe measurement periods.

On the other hand, the average number of hits in *T* during *Δ* is *CI*_*T*_/*I*_*M*_, so we can expect one hit on average in *T* after

(4) $$P=\frac{I_{M}}{{\mathrm{CI}}_{T}}$$

measurement periods; note that they eat up a total time of $P\Delta =\frac{1}{\gamma \phi I_{T}}$.

To see a hit in *T* before the measurement is affected by a triple hit in *M* on average, we should have something like *P* ⩽ *P*_*M*_, which is guaranteed if $\frac{I_{M}}{I_{T}}⩽3$. This is a very conservative estimate, however. To obtain the actual probability of failure *P*_*fail*_, i.e., of an unsafe measurement period within two consecutive target hits, we will assume that the number of hits in *M* and *T* follow a compound Poisson distribution with the same average hit rate per *μm* · *s*. This implies a rate *λ*_*M*_ = *C* per measurement period in *M*, and *λ*_*T*_ = *CI*_*T*_/*I*_*M*_ in *T*.

Recalling the geometric distribution of safe measurement periods with parameter *p* and the fact that the probability of no target hit within *k* measurement periods is $e^{-\lambda_{T}k}=e^{-C_{T}k}$, where we used the abbreviation *C*_*T*_ = *CI*_*T*_/*I*_*M*_, we find

$$P_{\mathrm{fail}}=\underset{k⩾0}{\sum }p{\left(1-p\right)}^{k}e^{-C_{T}k}=\frac{p}{1-\frac{1-p}{e^{C_{T}}}}=\frac{{\mathrm{pe}}^{C_{T}}}{e^{C_{T}}-1+p}\text{.}$$

Since the Poisson distribution of *H*_*M*_ implies *p* = *P{H*_*M*_ ⩾ 3} =  1 −  (1 + *C* + *C*^2^/2)*e*^−*C*^ = 1 − (1 + *C*′)*e*^−*C*^ with *C*′ = *C* + *C*^2^/2, we thus easily obtain

(5) $$P_{\mathrm{fail}}=\frac{(1-(1+C^{\prime })e^{-C})e^{C_{T}}}{e^{C_{T}}-(1+C^{\prime })e^{-C}}=\frac{1-(1+C^{\prime })e^{-C}}{1-(1+C^{\prime })e^{-C-C_{T}}}=\frac{1-(1+C+\frac{C^{2}}{2})e^{-C}}{1-(1+C+\frac{C^{2}}{2})e^{-C\overset{˙}{(}\frac{I_{M}+I_{T}}{I_{M}})}}\text{.}$$

Expression (5) for *P*_*fail*_ can be made as small as desired by choosing *C* ∈ (0, 2] sufficiently small, for all reasonable ratios *I*_*T*_/*I*_*M*_. For example, for *I*_*T*_ = *I*_*M*_/2, which is more than reasonable for all target circuits except for the inverter chain according to Table 2, we obtain *P*_*fail*_ < 0.01 for *C* = 0.2. For the inverter chain, Table 2 reveals *I*_*T*_ = *I*_*M*_/20, which yields *P*_*fail*_ < 0.1 for *C* = 0.2. Note that, according to (4), *C* = 0.2 leads to *P* = 5 measurement periods between two target hits on average. Given the quite conservative assumptions underlying our probabilistic analysis, we can hence finally conclude that our measurement architecture is indeed excellently suited for collecting statistically meaningful long-term data.

## 9 Conclusions

We provided an overview of the general approach for developing meaningful digital radiation failure models for digital asynchronous circuits in our FATAL project. We utilize a chain of 3D models and Spice models for model validation and calibration, along with radiation experiments utilizing a specifically designed FRad Chip that will be used for gathering radiation failure statistics in continuous long-term experiments.

We presented our choice of target circuits and on-chip measurement architecture of the FRad Chip, along with the results of the pre-fabrication analysis using our general FATAL approach. Key challenges are (i) distinguishing SETs from normal switching activity of the target circuits, (ii) providing reliable SET data acquisition in spite of radiation hits in the measurement infrastructure, and (iii) leaving as much of the die area available for the target circuits as possible. Rather than employing a rad-hard design, our architecture considers the measurement circuitry as additional target circuits, and hence allows to tolerate hits in the former by an architectural design that supports reliable fault detection based on a fault dictionary.

Our circuit architecture has been evaluated by means of elaborate fault-injection experiments based on detailed 3D models and Spice models of the FRad Chip, which were in turn validated and calibrated using micro-beam radiation experiments. For the latter, target circuits instrumented with high-speed analog-amplifiers have been used for analog SET recording. Together with a probabilistic analysis based on experimental cross-section data, the results of this evaluation allow us to predict that the FRad Chip will indeed serve its purpose as an effective means for the end-to-end validation of digital failure models.

The main focus in our future work is clearly on finalizing the design of the FRad Chip and manufacturing the ASIC. In addition, our research has also revealed the need for a refined understanding of the SET generation process and, consequently, refined Spice models, which we plan to pursue further. We also plan to leverage our combined simulation/experiment approach for studying the dependence of (transient) SEE impact and frequency on operating conditions like supply voltage and temperature in detail. This will be an interesting contribution to our general FATAL high-level modeling approach. Beyond the SET generation we are investigating SET propagation as well, and, specifically in the context of asynchronous circuit structures, we have recently come up with some encouraging findings.

## Figures and Tables

**Table 1:** Number of transistors for different architectures.

Architecture	Total no. of transistors
17-Inverter chain	34
33-Flip-flop chain	594
128-input NAND–NOR Tree	508
128-input XNOR tree	1016
35 C-element Van-Berkel elastic pipeline	490
35 C-element Weak-Feedback elastic pipeline	350
35 C-element conventional elastic pipeline	490
16-bit LFSR counter	440
32-bit LFSR counter	856
9-bit Up/down counter	187

**Table 2:** Hardware overhead analysis for measurement setup.

Architecture	No. of trans. for target circuits	No. of trans. for measurement circuits	Overhead factor (target circuit circuits as base)
Inverter chain	102	2086	20.451
Flip-flop chain	1782	2086	1.171
NAND–NOR tree	1524	2086	1.369
XNOR tree	3048	880	0.289
Elastic pipeline	1330	2086	1.568
All	7786	9224	1.185

**Table 3:** Operation of the LFSR counter in no-fault scenario.

Time (ns)	LFSR count	Actual count
0–10	4194310	1
10–50	0	0
50–60	4194310	1
60–70	12582922	2
70–80	29360146	3
80–90	62914594	4
90–100	130023490	5

**Table 4:** LFSR counter – SETs in XNOR gates and flip-flops.

Time (ns)	LFSR count	Actual count
*XNOR gate tapped between Q*_*1*_*and Q*_*2*_		
60–70	12582922	2
70–75	29360146	3
75–80	2936014**4**	**2325803548**
80–90	6291459**8**	**2325803549**

*Flip-flop with output Q*_*15*_		
60–70	12582922	2
70–80	29360146	3
80–90	629**47362**	**1647004572**
90–100	1300**89026**	**1647004573**

**Table 5:** Fault analysis of the up/down counter.

Time (ns)	Up/down count	Actual count
*C-element (with set) between the outputs Z*_*4*_*and Z*_*5*_		
65–70	111110000	5
70–75	000001111	5
75–80	1111**0**0000	**4**
80–85	0000**1**1111	**4**

*C-element (with rst) at output Z*_*5*_		
95–100	111110000	5
100–105	000001111	5
105–110	1111**0**0000	**4**
110–115	0000**1**1111	**4**

*C-element (with set) at output Z*_*8*_		
120–125	111110000	5
125–130	000001111	5
130–135	00000**000**1	**8**
135–140	11111**111**0	**8**

**Table 6:** Fault dictionary.

Observed syndrome				Location of faults	
*U*_1_	*U*_2_	*L*_1_	*L*_2_	Actual location	Interpretation
*No fault scenario*					
5	5	√	√	–	–

*Single fault scenario*					
6	5	+	√	*D*_1_	*D*_1_
4	6	√	√	*D*_2_	*D*_2_
5	4	√	+	*D*_3_	*D*_3_
∗	5	√	√	*U*_1_	*U*_1_
5	∗	√	√	*U*_2_	*U*_2_
5	5	*X*	√	*L*_1_	*L*_1_
5	5	√	*X*	*L*_2_	*L*_2_

*Double faults scenario*					
Location of all faults traceable					
Problematic triple fault scenarios					
∗	5	*X*	√	(*U*_1_, *L*_1_, *D*_1_)	(*U*_1_, *L*_1_)
∗	∗	√	√	(*U*_1_, *U*_2_, *D*_2_)	(*U*_1_, *U*_2_)
5	∗	√	*X*	(*U*_2_, *L*_2_, *D*_3_)	(*U*_2_, *L*_2_)

Problematic quadruple fault scenarios					
∗	∗	+	√	(*D*_1_, *D*_2_, *U*_2_, *U*_1_)	(*D*_1_, *U*_2_, *U*_1_)
6	∗	+	*X*	(*D*_1_, *D*_3_, *U*_2_, *L*_2_)	(*D*_1_, *U*_2_, *L*_2_)
∗	∗	*X*	√	(*D*_1_, *U*_1_, *U*_2_, *L*_1_)	(*U*_1_, *U*_2_, *L*_1_)
∗	6	*X*	√	(*D*_1_, *D*_2_, *U*_1_, *L*_1_)	(*U*_1_, *D*_2_, *L*_1_)
5	∗	*X*	√	(*D*_1_, *D*_2_, *U*_2_, *L*_2_)	(*D*_1_, *U*_2_, *L*_2_)
∗	5	*X*	*X*	(*D*_1_, *U*_1_, *L*_1_, *L*_2_)	(*U*_1_, *L*_2_, *L*_1_)
∗	∗	√	+	(*D*_2_, *D*_3_, *U*_1_, *U*_2_)	(*D*_3_, *U*_2_, *U*_1_)
∗	5	√	*X*	(*D*_2_, *D*_3_, *U*_1_, *L*_2_)	(*L*_2_, *U*_1_)
4	∗	√	*X*	(*D*_2_, *D*_3_, *U*_2_, *L*_2_)	(*D*_2_, *U*_2_, *L*_2_)
∗	∗	*X*	√	(*D*_2_, *U*_1_, *U*_2_, *L*_1_)	(*U*_1_, *U*_2_, *L*_1_)
∗	∗	√	*X*	(*D*_2_, *U*_1_, *U*_2_, *L*_2_)	(*U*_1_, *U*_2_, *L*_2_)
∗	∗	√	*X*	(*D*_3_, *U*_1_, *U*_2_, *L*_2_)	(*U*_1_, *U*_2_, *L*_2_)
5	∗	*X*	*X*	(*D*_3_, *U*_2_, *L*_1_, *L*_2_)	(*U*_2_, *L*_1_, *L*_2_)

## Footnotes

2 This work is supported by the Austrian Science Fund (FWF) under project numbers P21694 and P20529. For more information consult http://ti.tuwien.ac.at/ecs/research/projects/fatal/.

3 Due to the very long simulation times of the complex 3D device simulations and the huge amount of data recorded in our experiments, we are still refining our calibration. In fact, to increase the confidence in the 3D model, we will conduct additional measurement campaigns at the GSI microprobe facility, e.g., using heavy-ions with different energies.

4 The simple dependence in Fig. 4 would not hold if the timing parameters *T*_*α*_, *T*_*β*_ were also varied.

5 This can be resolved on a statistical basis, which is one of our future aims.

6 Given that *M* can also tolerate many triple and even higher-order faults, this is a very conservative assumption.
